# Subwavelength Bayer RGB color routers with perfect optical efficiency

**DOI:** 10.1515/nanoph-2022-0069

**Published:** 2022-03-30

**Authors:** Peter B. Catrysse, Nathan Zhao, Weiliang Jin, Shanhui Fan

**Affiliations:** E. L. Ginzton Laboratory, Department of Electrical Engineering, Stanford University, Stanford, USA; Department of Applied Physics, Stanford University, Stanford, USA; E. L. Ginzton Laboratory, Stanford University, Stanford, USA

**Keywords:** adjoint variable method, gradient descent optimization, ideal color separation, image sensors, perfect optical efficiency, subwavelength Bayer RGB color router

## Abstract

We introduce subwavelength color routers with perfect optical efficiency in a red-green-green-blue (RGGB) Bayer layout for solid state image sensors. This is the first demonstration of a subwavelength device concept that shows the full potential of color routing, i.e., perfect routing without loss of photons, with a broadband, polarization-independent, and angular robust response. As an example, we show a color router for 320 nm wide image sensor pixels, which are two times smaller than the smallest state-of-the-art pixels, that features perfect optical efficiency, i.e., no crosstalk between color channels, no reflections, and no leakage into non-photodetector regions, even though the pixel photodetectors are 2–3 times smaller than the wavelength of the incident light. Our color router outperforms all other color separation approaches and can replace the entire optical stack in solid state image sensors.

## 1 Introduction

Color is an essential functionality of image sensors in the visible range. This functionality is currently provided by color filters. Solid state image sensors incorporate a color filter array (CFA), comprised of color filters made from absorptive materials that are part of the optical stack above the pixel photodetectors [1, 2]. The fundamental limitation of the CFA approach is the inefficient usage of light incident on the image sensor. Photons that are absorbed by the color filters do not reach the photodetectors and therefore do not contribute to the detected signal.

**Figure 1:**
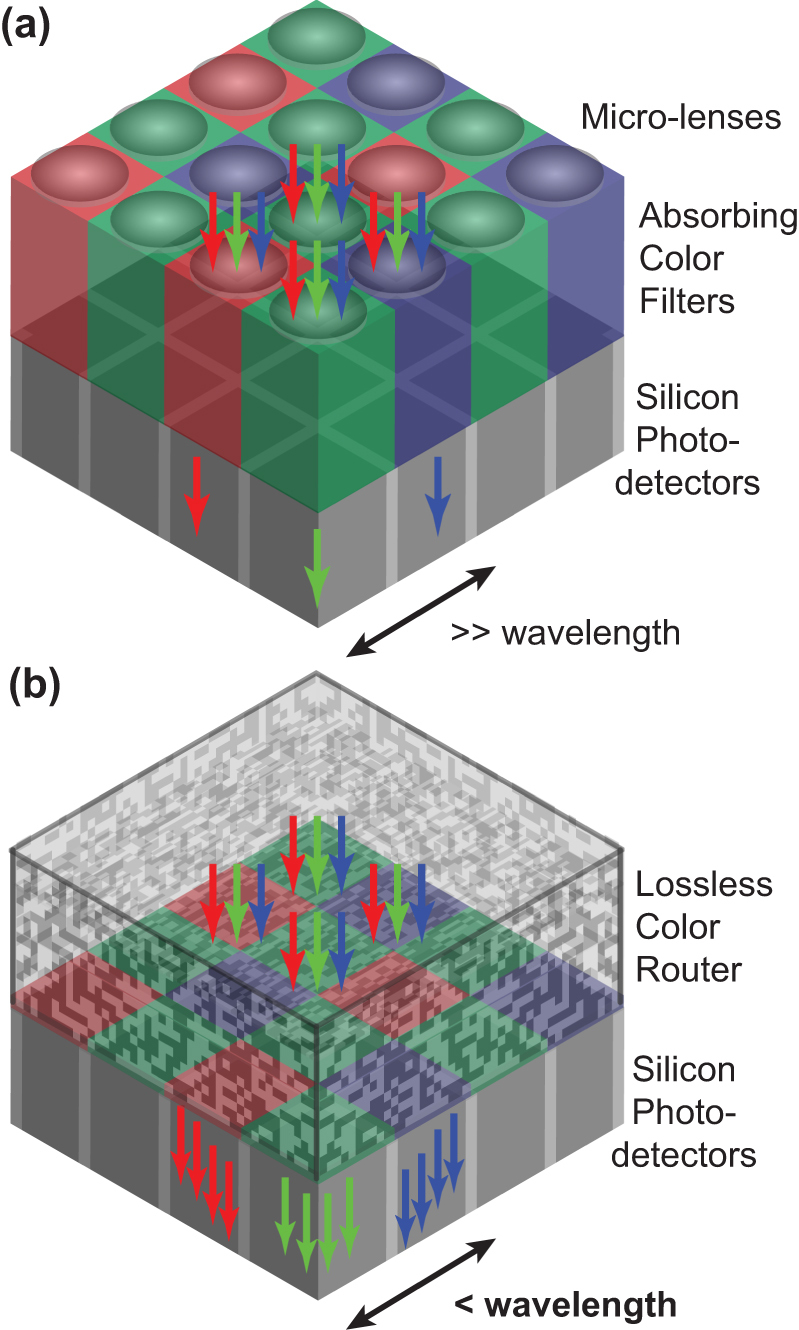
Subwavelength Bayer RGB color router. (a) Traditional color functionality in image sensors based on a CFA with red (R), green (G), and Blue (B) absorbing color filters in an RGGB Bayer mosaic layout and a micro-lens array on top of silicon photodetectors. (b) Lossless color router in a 2 by 2 pixel RGGB Bayer layout replaces both the CFA and the micro-lens array.

The most common CFA for image sensors is a periodic array with a red-green-green-blue (RGGB) Bayer kernel or unit cell (Figure 1(a)) [2, 3]. Each unit cell is placed on top of a 2 by 2 pixel photodetector array. As the pixel photodetectors continue to scale well below 1 μm in size [4], allowing more pixels and thus more image samples for a fixed image sensor format [5], [6], [7], [8], the scaling of the CFA kernel has not. In the latest generation CMOS image sensors, more than one pixel photodetector is placed under each of the R, G, G, and B color filters and the size of the Bayer kernel is at 3.2 μm well above 1 μm in size [9]. Bayer kernels with more than one pixel photodetector per color filter result in non-uniform sampling, which makes the demosaicing process more challenging as well as more prone to aliasing artefacts than the regular Bayer CFA [10, 11].

These observations on the fundamental and practical limitations of CFAs comprised of absorptive color filters suggest the need for a much more photon efficient approach to color separation in solid state image sensors. A color router is a device that *routes all incident light* *based on its color content*, i.e., different parts of the visible spectrum, *directly (without additional propagation) and without loss* to the silicon photodetector of the intended color channel.

While improved photon efficiency is important for any pixel size, it is critically important for CMOS image sensors with sub-micrometer pixels, since the photosensitive photodetector area decreases quadratically with pixel size. Moreover, other pixel components in the optical stack, such as the micro-lenses, have also been shown not to scale further as pixel size keeps shrinking [12]. Hence, the development of a color router with a subwavelength footprint that scales down in size with sub-micrometer pixels while improving photon efficiency constitutes an important advancement to further progress in CMOS image sensor technology. Recently, a number of lossless approaches for separating colors have been proposed [13], [14], [15], [16], [17], [18], [19], [20], [21]. None of these approaches, however, can operate as a color router with subwavelength footprint in a CMOS image sensor.

In this work, we introduce subwavelength color routers with perfect optical efficiency in a 2 by 2 pixel RGGB Bayer layout (Figure 1(b)). Our numerical optimization yields optical efficiencies exceeding 0.99, but additional increases in efficiency remain possible through further optimization. Hence, we refer to our concept for color separation as color routers with perfect optical efficiency. As an example of the ultimate performance that a color router can achieve without compromising on its ability to scale, we show a device for 320 nm wide image sensor pixels, which are two times smaller than the smallest state-of-the-art pixels currently found in CMOS image sensors [22].

## 2 Color router design strategy

Our color router has to be considered in the context of the image sensor pixel geometry to create a device that can operate in solid state image sensors. Color routers are intended as direct replacements for the conventional optical stack, which is comprised of the micro-lenses and absorbing color filters placed on the silicon photodetectors of the image sensor pixels (Figure 1(a)). Therefore, we focus for the color router design on the optical stack region above the pixel photodetectors (Figure 1(b)). We use the volume that is currently taken up by these color filters and micro-lenses and we integrate the color router on the silicon substrate that contains the pixel photodetectors. We apply CMOS compatible dielectric materials only and avoid structures with air gaps in the color router design to maintain mechanical stability and integrity.

To design a subwavelength color router in a 2 by 2 pixel RGGB Bayer layout, we consider a design volume with a height of 2 μm and a 640 nm by 640 nm footprint, which is subwavelength for the center wavelength (650 nm) of the red color channel. The parameters of the design are a set of design elements that occupy this volume and are assumed here to be 40 nm tall and 10 nm by 10 nm wide. The dielectric constant of each element is taken to be between 2.1 and 7, which represent silica and titanium dioxide, respectively, both of which are effectively lossless at the visible wavelengths of interest here [23]. The design region is integrated on top of the 2 by 2 pixel kernel with the silicon photodetectors of the R, G, and B color channels. A subwavelength size color router design of 640 nm therefore enables a 320 nm pixel size, which is a few generations ahead of current state-of-the-art image sensor pixel technology [22]. The photosensitive silicon photodetectors of these pixels are even smaller (260 nm) to allow for pixel deep trench isolation (DTI) regions, i.e., silicon oxide regions that surround the photodetectors and prevent optical and electrical crosstalk in the silicon substrate of back-side illuminated image sensors [24, 25]. This means that the photodetectors, which are the color routing target regions, are 2–3 times smaller than the wavelength of the incident light.

We quantify the performance of the color router design by calculating the optical efficiency for each color channel [19, 26, 27], which is the fraction of the total optical power incident on the entire device that is routed to the photodetector of the intended color channel. In addition, we measure the optical crosstalk between the color channels [12, 19, 27]. This is the fraction of the incident optical power for the intended color channel that is improperly routed to the photodetectors of the adjacent color channels. Lastly, we measure the light reflected from the device as well as the light incident on non-photosensitive areas of the silicon surface [19]. That way we account for all light incident on the entire device, which allows us to establish how close to ideal or perfect the performance of the subwavelength color router is.

Our goal for the color router design is to significantly improve the efficiency of current color pixel technology and to ultimately achieve routing of all incident light based on its color content with perfect optical efficiency. The design strategy for realizing this goal consists of exploiting the degrees of freedom in the design region. If we assign one of two different dielectric materials to each design element, the resulting search space will have an enormous number of degrees of freedom and a brute-force search for suitable structures is not practical. Instead, we employ an efficient computational design strategy based on the adjoint variable method and gradient descent optimization to take advantage of the very large number of degrees of freedom [19, 28], [29], [30], [31].

We perform first-principles calculations of the color router response to incident light using a rigorous coupled wave analysis (RCWA) solution to Maxwell’s equations [32]. To optimize the design parameters of the device, the gradients are provided by the adjoint variable method, which is implemented for the RCWA solver [33]. The optimizer is based on a quasi-Newton method called the method of moving asymptotes [34]. In this strategy, we aim at a systematic optimization of an objective function that maximizes the optical power intended for each photodetector (R, G, B) based on the spectral content (color) of the incident light. Our objective function is *F* = ∑_*i*=R,G,B_[*α*OE(*i*) – (1 − *α*)∑_*j*≠*i*_OX(*j*,*i*)], where OE(*i*) is the optical efficiency of the *i*th color channel, OX(*j*,*i*) is the optical crosstalk from the *j*th color channel into the *i*th color channel, and *α* is a weighting factor with a value between 0 and 1.

## 3 Results

**Figure 2:**
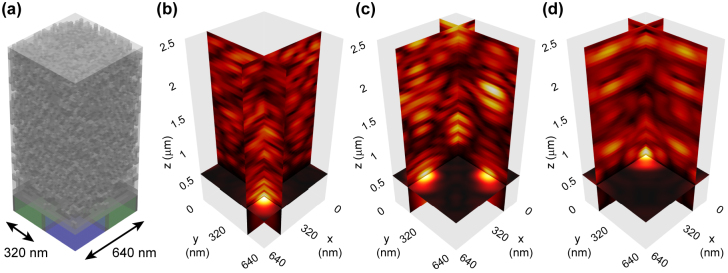
Subwavelength Bayer RGB color router with perfect optical efficiency. (a) Optimized RGB color router for image sensor pixels with 320 nm pixel pitch and a subwavelength device size of 640 nm. Dark and light cubes represent design elements made of titanium dioxide (*ε* = 7) and silica (*ε* = 2.1), respectively. (b)–(d) Three-dimensional electric field distributions for the B, G–G, and R color channels at the operating wavelengths of 450, 550, and 650 nm, respectively.

To demonstrate a subwavelength Bayer RGB color router that achieves ideal lossless color routing, we run a gradient-based optimization for spectral colors that correspond to the central wavelengths (450, 550, 650 nm) of the B, G, R color channels. Figure 2(a) shows the optimized color router device structure on top of the silicon photodetectors of the R, G, and B color channel pixels. Dark cubes represent design elements made of titanium dioxide, while lighter cubes represent design elements filled with silica. Figure 2(b)–(d) show the three-dimensional electric field distributions at the operating wavelengths of 450, 550, and 650 nm, respectively. The two-dimensional vertical *xz*, *yz* and horizontal *xy* cross-sections in Figure 2(b)–(d) intersect at the B, G–G, and R pixel photodetector locations, respectively. They clearly demonstrate the ability of the subwavelength color router design to route incident light based on its color content directly, i.e., without need for any additional propagation beyond the device, to the photodetectors of the intended R, G, and B color channels.

Table 1 summarizes the performance of the optimized RGB color router. The routing is perfect with an optical efficiency >0.99 for all color channels. The optical crosstalk between channels is effectively zero (<0.01). The light reflected by the subwavelength color router, as well as the light incident on the non-photosensitive DTI regions of the underlying pixel structures are negligible. Hence, the device functions not only as an ideal color router, but also acts as a perfect anti-reflection coating and replaces the functionality of a micro-lens. The latter is significant since micro-lenses cease to function at subwavelength scale [12].

**Table 1:** Subwavelength Bayer RGB color router with perfect optical efficiency for the R, G, B color channels (bold values) and with no crosstalk, no reflections and no light leaking into non-photosensitive DTI regions.

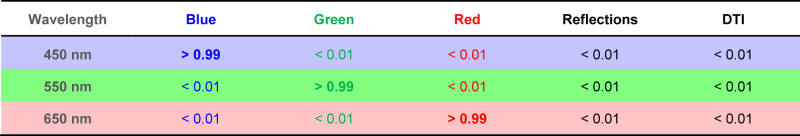

The subwavelength color router we designed here is more than 2 times smaller than the smallest CFA kernels with absorbing filters that have been demonstrated [9, 35]. To evaluate the optical efficiency against a CFA approach, we compare with the transmittance of the corresponding ideal color filter. We note, however, that such a corresponding color filter does not exist in practice for subwavelength pixels, since color filters do not scale to sub-micrometer size [36, 37]. While current larger color filters have peak transmission values in the 0.80–0.90 range [38], they only transmit the light that is directly incident on them, i.e., 25% (R, B) or 50% (G) of all the light incident on the entire RGGB Bayer kernel. Hence, if ideal color filters existed and could maintain their transmission values at sub-micrometer scale, we would obtain corresponding optical efficiencies of around 0.2 (R, B) and 0.4 (G). A color router approach can therefore improve optical efficiency over the corresponding ideal color filters by 2.5–5 times, despite being subwavelength in size.

To consider fabrication constraints on color router implementation, we use the design element width as a measure for the minimum feature size in an implementation. To assess the effect of the minimum feature size on the color router performance, we increase the element design width two-fold. We find that subwavelength color routers based on such larger design elements can still be optimized to achieve optical efficiencies >0.99 for all color channels with effectively zero (<0.01) optical crosstalk, reflections and light incident on the non-photosensitive DTI regions.

**Figure 3:**
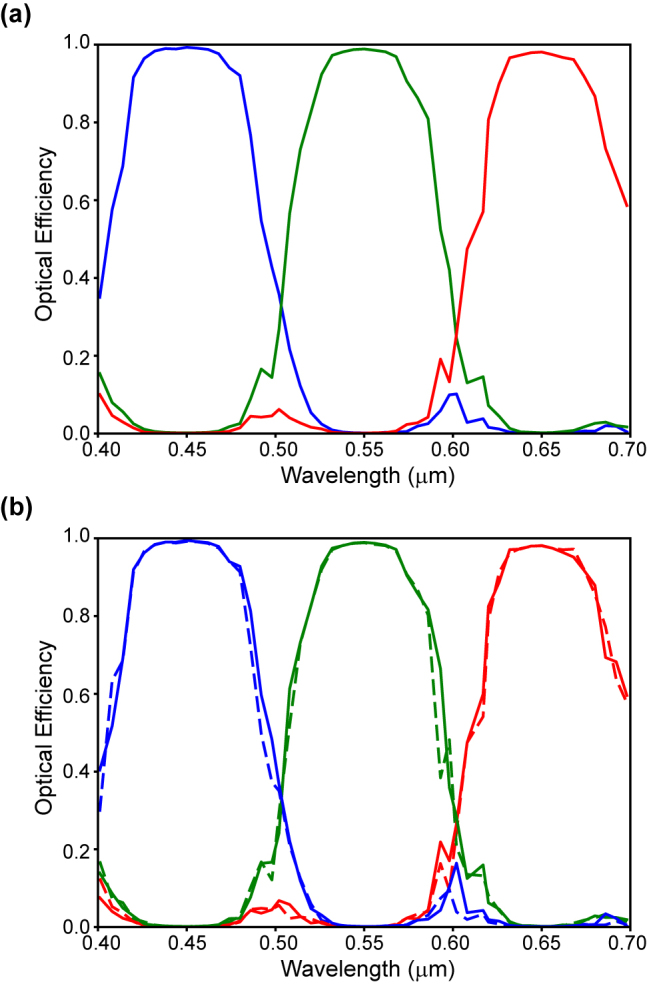
Broadband and polarization independent response of a subwavelength Bayer RGB color router. (a) Broadband spectral response for the R, G, B color channels with perfect (>0.99, 0.99) B, G and near-perfect (>0.98) R peak optical efficiencies. (b) Polarization independent response with near-identical spectral curves for the *x*- and *y*-polarization (solid and dashed curves) components.

We also analyze the spectral, polarization, and angular response and demonstrate that the performance of color routers based on lossless materials remains robust for incident imaging light conditions. The light incident on solid state image sensors is spectrally broadband. For color imaging, this spectrum covers the visible wavelength range (400–700 nm). To capture the spectral information that allows images to be rendered in full color for the human visual system, a color router has to perform over this range. Figure 3(a) shows the results for a color router that has been optimized for broadband spectral response with center wavelengths (450, 550, 650 nm) for the B, G, R color channels. The spectral response for each of the color channels features a spectral bandwidth of ≈100 nm full width at half maximum and covers the entire visible wavelength range. The color router achieves perfect peak optical efficiencies (>0.99, 0.99) for the B, G color channels, and near-perfect optical efficiency (>0.98) for the R color channel.

The light incident on image sensors is generally not polarized and absorbing color filters are polarization independent. Hence, we have to analyze the polarization response of the color router approach. Figure 3(b) shows the spectral response of the *x*- and *y*-polarization (solid and dashed curves) components for the optimized color router shown in Figure 3(a). This design was optimized without enforcing any symmetry on the device structure, yet the curves for the polarizations are nearly identical. This shows that the broadband spectral response of the color router is also polarization independent.

Finally, the light incident from an imaging lens comprises a range of incidence angles, i.e., it has an angular extent, which is characterized by the f-number (f/#) of the lens [5, 39]. For image sensors with the smallest pixels, typical imaging lenses are in the f/2.8–1.8 range, which corresponds to ±10–16 degrees of angular extent. Within this extent, the optical efficiency has to remain high, so that the performance of the color router remains ideal under imaging illumination. Typical absorbing color filters exhibit a degree of robustness in their angular response [38]. We verified that the optical efficiency of a color router can be maintained at near-perfect levels, above 0.98 (0.98), over ±10 (16) degree range covered by an f/2.8 (f/1.8) imaging lens.

## 4 Discussion

We have introduced subwavelength color routers with perfect optical efficiency in an RGGB Bayer kernel layout for CMOS image sensors. This is the first time a subwavelength device is shown that achieves the full potential of color routing, i.e., perfect routing without loss of photons, with a broadband, polarization-independent, angular robust response.

As an example, we showed a subwavelength color router for 320 nm image sensor pixels, which are two times smaller than the smallest state-of-the-art pixels found in CMOS image sensors. The optimized device routes incident light based on color content to the R, G, and B channel photodetectors with perfect optical efficiencies. This means that there is no crosstalk into photodetectors of the other channels, no reflection, and no light leaking into non-photodetector regions, despite the photodetectors being 2–3 times smaller than the wavelength of the incident light. We note, however, that color routers with perfect optical efficiency can be achieved for current larger pixel sizes as well.

A color router outperforms and replaces all components in the optical stack, including color filters, micro-lenses and anti-reflection coatings, of a conventional pixel at once. We also showed that perfect optical efficiencies are maintained for broadband spectral response, polarization independent response and angular response. These are all essential features for using color routers in solid state image sensors. Our demonstration here is a few generations ahead of current image sensor technology and demonstrates that further scaling while continuously improving optical efficiency is possible. With this approach, pixels can have a 2.5–5 times smaller surface area and yet their photodetectors will receive the same amount of light as correspondingly larger pixels making use of a CFA with absorbing RGB color filters.

The fabrication of subwavelength color routers will require advanced nanolithography methods, such as those used in state-of-the-art multilayer semiconductor manufacturing with feature sizes in the sub-100 nm range. Nanolithography with feature sizes down to ∼10–20 nm has already been shown with next-generation lithography tools [40].

Due to their unique combination of functionalities, ultimate performance and scaling ability, subwavelength color routers constitute an important advancement in photon efficient color functionality necessary for the development of future image sensor technologies. More broadly, our color routers point to the possibility of light routing at the subwavelength scale with perfect photon efficiency for light properties in general. Our approach thus forms a very general framework for detecting and analyzing properties of light at the subwavelength scale without any photons being lost. This may open up opportunities in a variety of fields where nanophotonics plays a prominent role and where reliable measurement of light properties depends on collecting most, if not all, of the available photons.
